# Rapid Effect of Nitrogen Supply for Soybean at the Beginning Flowering Stage on Biomass and Sucrose Metabolism

**DOI:** 10.1038/s41598-019-52043-6

**Published:** 2019-10-29

**Authors:** Hongli Zhou, Xingdong Yao, Qiang Zhao, Wei Zhang, Bo Zhang, Futi Xie

**Affiliations:** 1 Soybean Research Institute, Shenyang Agricultural University, Shenyang, China; 2 Soybean Research Institute, Jilin Academy of Agricultural Sciences, Changchun, China; 3 Virginia Tech Department of Crop, Soil and Environmental Sciences, Blacksburg, VA USA

**Keywords:** Plant physiology, Plant development

## Abstract

Nitrogen application at the beginning flowering stage (R1 stage) increased the soybean grain yield, however, the rapid effect of enriched nitrogen at R1 growth stage on soybean dry matter accumulation and sugar metabolism is still unclear. Continuous high nitrogen (CHN), Continuous low nitrogen (CLN), Enriched nitrogen supply at R1 stage (ENS) treatments were applied on two soybean cultivars (Liaodou11, Liaodou14), to investigate the effect of enriched nitrogen on plant biomass accumulation and sucrose metabolism. After 12 h of ENS treatment, the root/shoot rate of both cultivars were lower than that of CLN, but at 24 h it was no significant difference between ENS and CLN. Enriched N at R1 stage, soybean kept a balance of sucrose synthesis and decomposition in leaf by affecting sucrose synthetase (SS) and sucrose phosphate synthase (SPS) activities. Under N limitation condition the plant dry matter accumulation supported root growth priority. Enriched N at R1 stage resulted in the rapid shoot biomass accumulation. In high yield cultivar, the shoot growth was priority to root growth, the common yield cultivar was on the contrary. Our result suggest that enrich N at R1 stage resulted in the accumulation of biomass in shoot rapidly.

## Introduction

Nitrogen is an essential element for soybean (*Glycine max* L.)^1–4^. Nitrogen is critical for high grain yield^2,4,5^, by improving the leaf area, biomass^4^, grain-filling rate^6^, and translocation of stem non-structural carbohydrates^7^. The time of N application is one of the determinant factor to maximize the grain yield, which influenced the number of flowers^2,8^. Beginning flowering stage (R1) is an important stage while soybean convert from vegetative growth to reproductive growth, applying N at R1 stage prolonged the functional period of leaf, improved the photosynthetic capacity and grain yield^9,10^.

In higher plant, there are two major metabolic pathways of photosynthesis primary products, one is chloroplast starch synthesis, another is cytoplasmic sucrose synthesis^11–13^. Nitrogen regulated sugar metabolism^14^. Proper nitrogen application increased the fructose content in tomato^15^. Nitrogen application increased the soluble sugar content and sucrose content in grain^16^. Under high nitrogen condition, sucrose provided the energy and carbon skeletons, and led to the reduction of organic acid amounts and sustained phosphoenolpyruvate utilization^17,18^. Wang *et al*.^19^ considered that as the increment of nitrogen application, the content of soluble sugar increased at first and then decreased, and the contents of soluble sugar and sucrose were increased by proper nitrogen application.

In the sucrose-to-starch pathway, sucrose synthetase (SS) is the first step enzyme^6,11^. Chen *et al*.^20^ found that sucrose synthase played a key role of sucrose post-unloading pathways. Sucrose phosphate synthase (SPS) is key enzyme of sucrose rapid synthesis, which reduces the concentration of monosuccharide^21,22^. SPS was regulated by calcium, metabolites and reversible protein phosphorylation, no matter in photosynthetic nor nonphotosynthetic tissues^23^. Nitrogen promoted sucrose synthesis in leaf, which benefited for the increment of sucrose phosphate synthase and sucrose synthase activities^24^. At the early growth stage of sugar beet, nitrogen decreased the activities of sucrose synthase and sucrose phosphate synthase, high nitrogen was not benefit for sugar accumulation^25,26^.

Sucrose as a signal molecule influences plant growth by regulating the level of gene expression^27^. Liu *et al*.^28^ demonstrated that sucrose synthesis was closely related with *DcSus1*, *DcSus2*, and *DcSus3*. In cucumber, overexpression of *CsSPS4* led to the priority of sugar transport, in carbon metabolism, and suppression of *CsSPS4* promoted starch accumulation^22^. Under low N condition, SPS gene expression and SPS activity were higher than those under high N condition^7^, and the higher *OsSPS1* gene expression and SPS activity had positive correlations with the number of flower and grain yield^22^. But sucrose-phosphate synthase II transcript level could not be used as high-yield cultivar indicator^29^.

The N application affected the carbon metabolism and dry mass accumulation complicatly^8,30–33^. N application at flowering stage influenced the reproductive growth and grain yield significantly^4,9,34^. It have been proved that applying N at R1 stage significantly increased the grain yield of two soybean cultivars with different yield potentials^35^. However, the rapid effect of enrich N on biomass and carbon metabolism was not clear. As the growth of soybean at R1 stage have great significance for the reproductive growth^35^. To investigate the cause of yield increase, the rapid effect of enriched nitrogen at R1 growth stage on soybean dry matter accumulation, different kinds of sugar contention and sucrose metabolism have been measured. In this study, two soybean cultivars with different yield potentials and three N treatments (continuous high nitrogen treatment; continuous low nitrogen; enrich nitrogen supply at R1 stage treatment) were used, the biomass, soluble sugar, sucrose and starch content, SS activity, SPS activity and their relative gene expression (*GmSPS, GmSS1, GmSS5*) were measured to investigate the short-term sucrose metabolism and dry matter accumulation after enrich N at R1 stage.

## Result

### Response of dry mass to enriched N application at R1 stage

**Figure 1:**
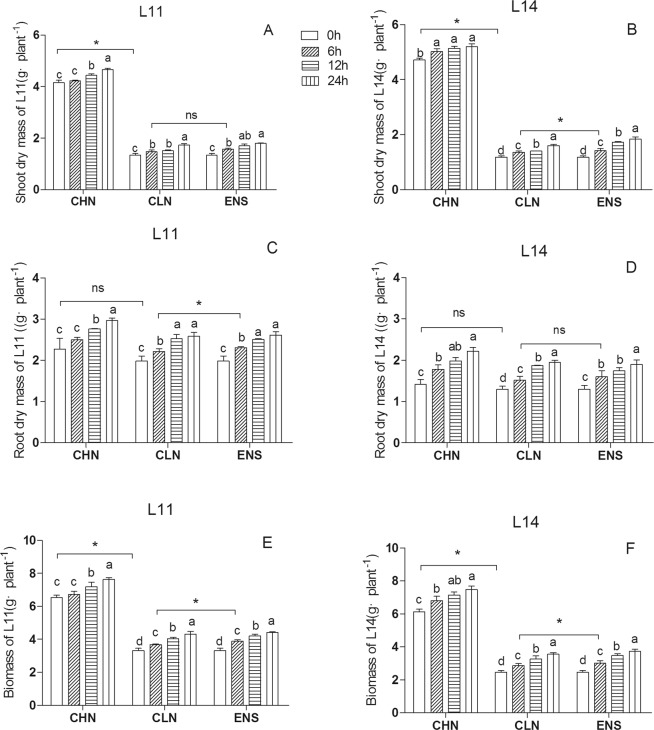
Shoot dry mass, root dry mass and total biomass of CV. L11 (**A**,**C**,**E**) and CV. L14 (**B**,**D**,**F**). Values are the means of the three replicates. STD errors are the standard deviation of three replicates (p < 0.05). *Means at one sampling time, there were significant differences between two treatments (p < 0.05). ns means at one sampling time, there were no significant differences between two treatments (p > 0.05).

Under CHN treatment, the shoot dry mass of CV.L11 was increased significantly from 0 h to 12 h, while high yield CV.L14 was increased significantly from 0 h to 6 h (P < 0.05) (Fig. 1A). Under CLN and ENS treatments, the shoot dry mass of two cultivars were increased significantly from 0 h to 6 h (P < 0.05) (Fig. 1A,B). The shoot dry mass of CV.L11 after 6 h of ENS treatment have no significant difference with CLN, however, the shoot dry mass of CV.L14 after 6 h of ENS treatment was significantly higher than that of CLN. Under CHN treatment, the root dry mass of CV.L11 was increased significantly from 0 h to 12 h, however the high yield CV.L14 was increased significantly from 0 h to 6 h (P < 0.05) (Fig. 1C,D). The root dry mass of CV.L11 after 6 h of ENS treatment was significantly higher than that of CLN, however, the root dry mass of CV.L14 after 6 h of ENS treatment have no significant difference with CLN. Under CHN treatment, the total dry mass of CV.L11 was increased significantly from 0 h to 12 h (Fig. 1E), but CV.L14 was increased significantly from 0 h to 6 h (P < 0.05) (Fig. 1F). Under CLN and ENS treatments, the total dry mass of both cultivars were increased significantly from 0 h to 6 h (P < 0.05) (Fig. 1E,F).

**Figure 2:**
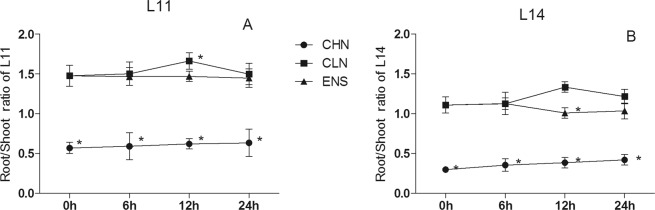
The root/shoot ratio of CV. L11 (**A**) and CV. L14 (**B**). Values are the means of the three replicates. STD errors are the standard deviation of three replicates (p < 0.05). *Means at one sampling time, CHN or CLN treatment were significant higher/lower than ENS treatment.

In both cultivars, the root/shoot ratio of CHN was significantly lower than that of CLN and ENS (P < 0.05) (Fig. 2A,B). The root/shoot ratio after 12 h of CLN treatment was significantly higher than that of CHN and ENS (P < 0.05) (Fig. 2A,B). After 0 h, 6 h and 24 h, there were no significant differences between CLN and ENS in both cultivars (Fig. 2A,B). The root/shoot ratio of high yield CV.L14 under CHN, CLN and ENS treatments were always lower than that of CV.L11 (Fig. 2A,B).

### Soluble sugar, sucrose and starch in response to enrich N application at R1 stage

**Figure 3:**
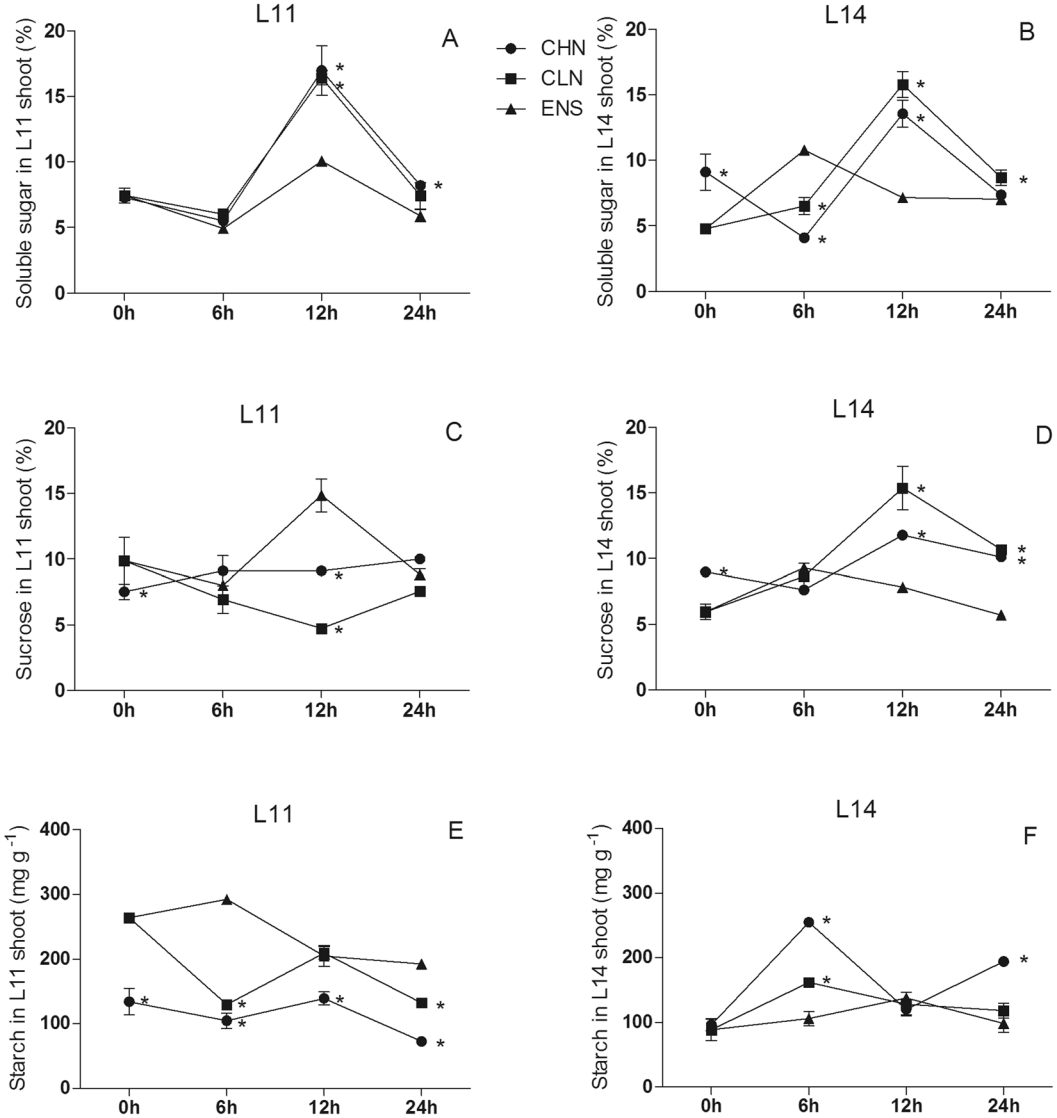
The content of soluble sugar, sucrose and starch in the leaf of CV. L11 (**A**,**C**) and CV. L14 (**B**,**D**). Values are the means of the three replicates. STD errors are the standard deviation of three replicates (p < 0.05). ^*^Means at one sampling time, CHN or CLN treatment were significant higher/lower than ENS treatment.

The soluble sugar content of CV.L14 shoot after 6 h of ENS treatment was 164% and 65% higher than those of CHN and CLN, respectively (Fig. 3B), but there was no significant difference among the three treatments in CV.L11(Fig. 3A). The shoot soluble sugar content of both cultivars after 12 h of ENS treatment was significantly higher than those of CHN and CLN (P < 0.05) (Fig. 3A,B). The sucrose content in CV.L11 shoot after 12 h of ENS treatment was significantly higher than those of CHN and CLN, and CHN was significantly higher than CLN (Fig. 3C). But in high yield CV.L14, the sucrose content of ENS treatment was significantly lower than those of CHN and CLN, and CHN was significantly lower than CLN (P < 0.05) (Fig. 3D). The starch content in CV.L11 shoot after 0 h, 12 h and 24 h of CHN treatment were lower than that of CLN treatment (Fig. 3E). However, in CV.L14 shoot, the starch content of CHN treatment were higher than that of CLN treatment after 6 h and 24 h (Fig. 3F). The starch content in CV.L11 shoot after 6 h of ENS treatment was significantly higher than those of CHN and CLN treatments (P < 0.05) (Fig. 3E). Conversely, the starch content in CV.L14 shoot after 6 h of ENS treatment was significantly lower than those of CHN and CLN treatments (P < 0.05) (Fig. 3F).

**Figure 4:**
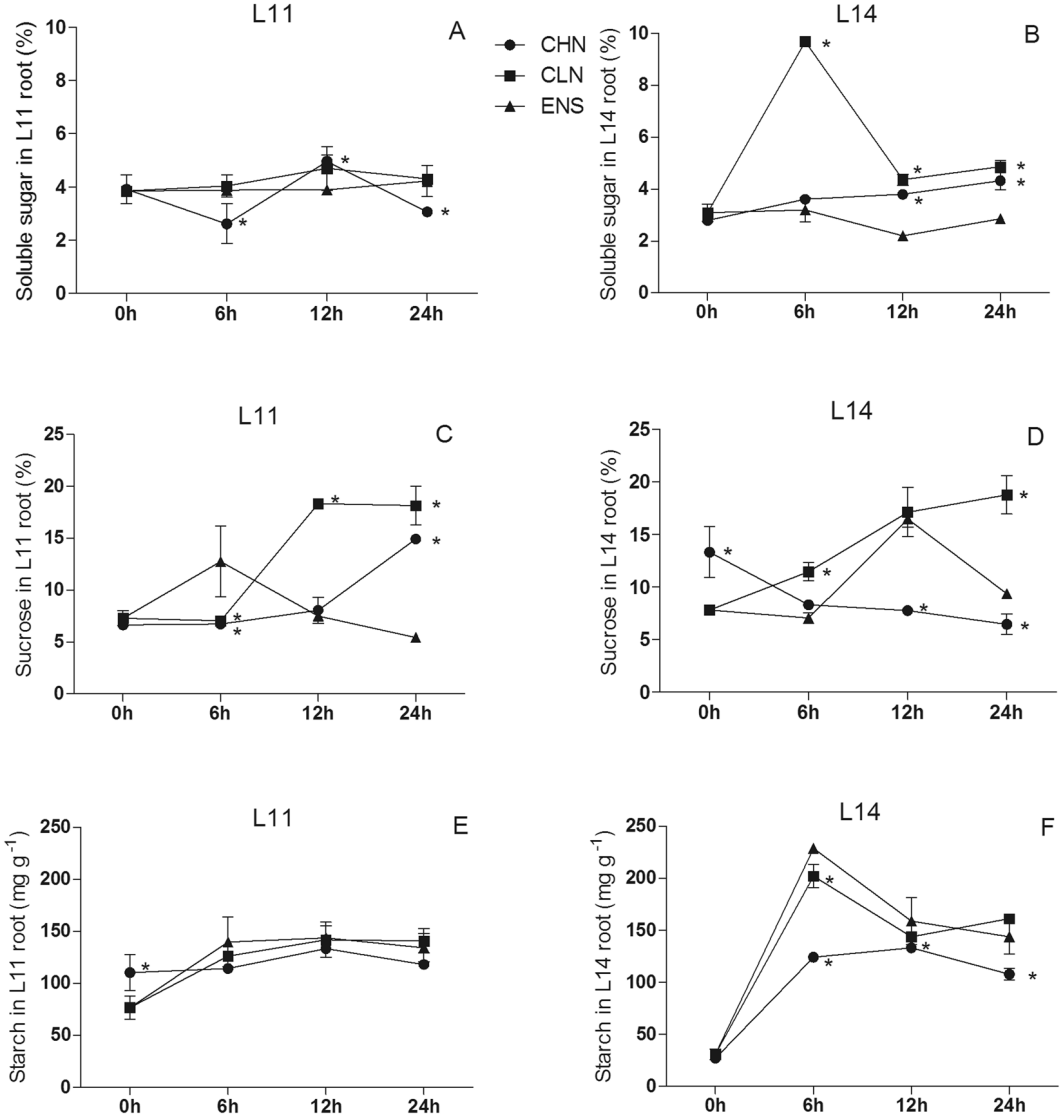
The content of soluble sugar, sucrose and starch in the root of CV. L11 (**A**,**C**) and CV. L14 (**B**,**D**). Values are the means of the three replicates. STD errors are the standard deviation of three replicates (p < 0.05). *Means at one sampling time, CHN or CLN treatment were significant higher/lower than ENS treatment.

The soluble sugar content of CV.L11 root after 12 h had no significant difference between CLN and CHN treatments, however, both treatments were significantly higher than that of ENS (P < 0.05) (Fig. 4A). The root soluble sugar content in CV.L14 of ENS treatment was always lower than those of CHN and CLN (Fig. 4B). The root sucrose content in CV.L11 after 6 h of ENS treatment was significantly higher than those of CHN and CLN treatments (P < 0.05) (Fig. 4C), but in CV.L14, the root sucrose content of ENS treatment was 38% lower than that of CLN treatment (Fig. 4D). The root sucrose content in CV.L11 after 12 h of ENS treatment was significantly lower than that of CLN treatment (P < 0.05) (Fig. 4C). In CV.L14 root, there were no significant differences of sucrose content between ENS and CLN treatments, but they were significantly higher than that of CHN treatment (Fig. 4D). After 6 h, 12 h and 24 h under CHN, CLN and ENS treatments, there were no significant differences of starch content in CV.L11 root (Fig. 4E). The starch content in CV.L14 root after 6 h, 12 h and 24 h of ENS and CLN treatments were all higher than that of CHN treatment, while between ENS and CLN treatments, there were no significant differences except the 6 h (Fig. 4F).

### SS activity and SPS activity in leaf and root

**Figure 5:**
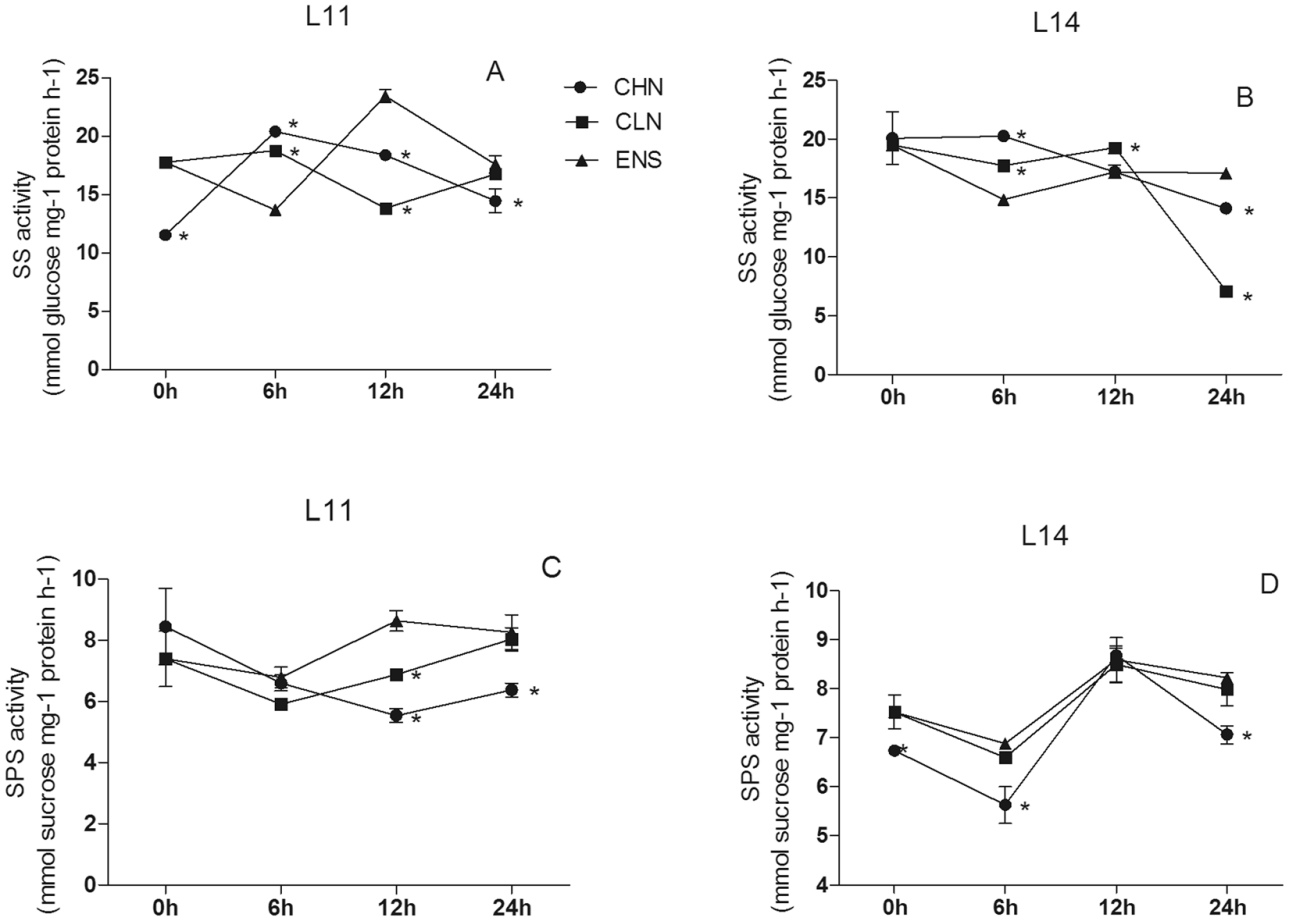
The SS and SPS activities in the leaf of CV. L11 (**A**,**C**) and CV. L14 (**B**,**D**). Values are the means of the three replicates. STD errors are the standard deviation of three replicates (p < 0.05). *Means at one sampling time, CHN or CLN treatment were significant higher/lower than ENS treatment.

The SS activity in CV.L11 leaf after 12 h of ENS treatment was significantly higher than those of CHN and CLN treatments (P < 0.05) (Fig. 5A), however there were no significant differences among CHN, CLN and ENS treatments in high yield CV.L14 (Fig. 5B). The SS activities in CV. L11 leaf after 24 h of ENS and CLN treatments were significantly higher than that of CHN treatment (P < 0.05) (Fig. 5A), however, in the high yield CV. L14, the SS activity of ENS treatment was significantly higher than those of CHN and CLN treatments (P < 0.05) (Fig. 5B). The SPS activity in CV.L11 leaf after 12 h of ENS treatment was 55% and 25% higher than those of CHN and CLN treatments (Fig. 5C), however there were no significant differences in the high yield CV.L14 leaf among CHN, CLN and ENS treatments (Fig. 5D). The leaf SPS activities in both cultivars after 24 h of ENS and CLN treatments were significantly higher than that of CHN treatment (Fig. 5C,D).

**Figure 6:**
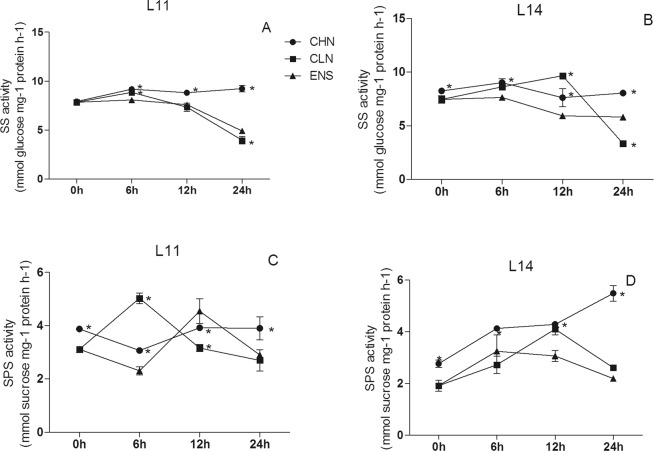
The SS and SPS activities in the root of CV. L11 (**A**,**C**) and CV. L14 (**B**,**D**). Values are the means of the three replicates. STD errors are the standard deviation of three replicates (p < 0.05). *Means at one sampling time, CHN or CLN treatment were significant higher/lower than ENS treatment.

After 0 h, 6 h, 12 h and 24 h under ENS treatment, the SS activity in CV.L11 root had no significant differences with CLN, but they were significantly higher than that of CHN treatment (Fig. 6A). The SS activity in high yield CV.L14 root after 12 h of ENS treatment was significantly lower than those of CHN and CLN treatments, and CHN treatment was significantly lower than that of CLN treatment (P < 0.05) (Fig. 6B). The SS activity in the high yield CV.L14 root after 24 h of ENS treatment was significantly higher than that of CLN treatment (P < 0.05) (Fig. 6B). The root SPS activities in both cultivar after 0 h and 24 h of CHN treatment were significantly higher than those of CLN and ENS treatments (P < 0.05) (Fig. 6C,D). The SPS activity in CV.L11 root after 6 h of CLN treatment was 63% and 118% higher than those of CHN and ENS treatments (Fig. 6C), however the SPS activity of CHN treatment was 51% and 26% higher than those of CLN and ENS treatments in the high yield CV.L14 (Fig. 6D). The SPS activity in CV.L11 root after 12 h of ENS treatment was significantly higher than those of CHN and CLN treatments (P < 0.05) (Fig. 6C). On the contrary, the SPS activity in the high yield CV.L14 root of ENS treatment was significantly lower than those of CHN and CLN treatments (P < 0.05) (Fig. 6D).

### The expression of SS and SPS genes in leaf and root

**Figure 7:**
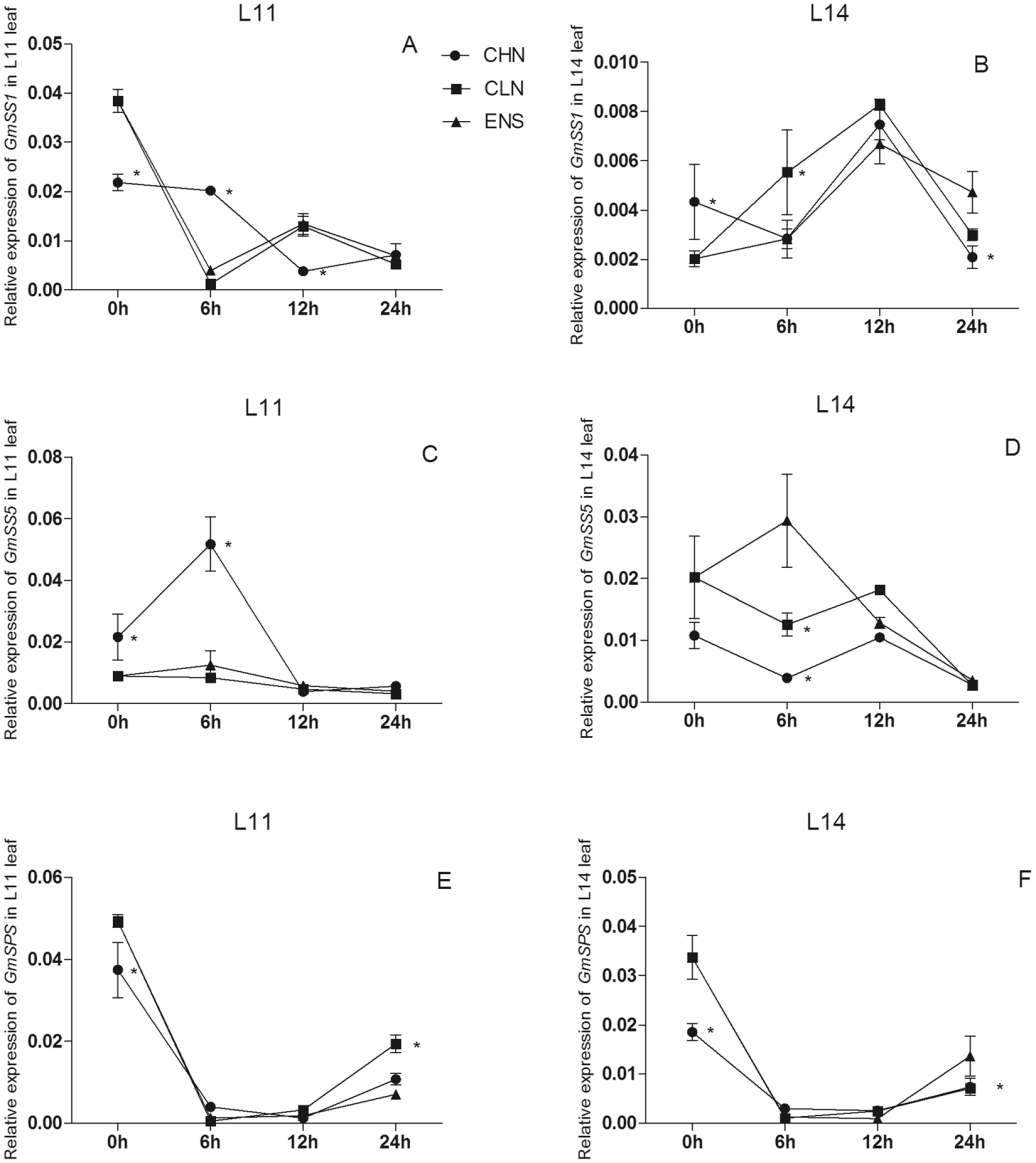
The expression of *GmSS1, GmSS5, GmSPS* in the leaf of CV. L11 (**A**,**C**) and CV. L14 (**B**,**D**). Values are the means of the three replicates. STD errors are the standard deviation of three replicates (p < 0.01). *Means at one sampling time, CHN or CLN treatment were significant higher/lower than ENS treatment.

The expression of *GmSS1* in CV.L11 leaf after 6 h of CHN treatment was significantly higher than those of CLN and ENS treatments (Fig. 7A), however in the high yield CV.L14, CLN treatment was significantly higher than CHN and ENS treatments (P < 0.05) (Fig. 7B). The expression of *GmSS1* have no significant differences among CHN, CLN and ENS treatments in L11 leaf after 24 h (Fig. 7A). The expression of *GmSS1* in the high yield CV.L14 after 24 of ENS treatment was 126% and 57% higher than CHN and CLN treatments (Fig. 7B). After 0 h, 6 h, 12 h and 24 h, the expression of *GmSS5* in leaf of both cultivars had no significant difference between ENS and CLN treatments (Fig. 7C,D), however, the expression of *GmSS5* in CV. L14 leaf after 6 h of ENS treatment was 133% higher than that of CLN treatment (Fig. 7D). After 6 h and 12 h, there were no significant differences in the *GmSPS* expression of 2 cultivars among CHN, CLN and ENS treatments (Fig. 7E,F). The *GmSPS* expression in CV.L11 leaf after 24 h of ENS treatment was significantly lower than those of CHN and CLN treatments (P < 0.05) (Fig. 7E). Conversely, the *GmSPS* expression in the high yield CV.L14 leaf of ENS treatment was significantly higher than those of CHN and CLN treatments (P < 0.05) (Fig. 7F).

**Figure 8:**
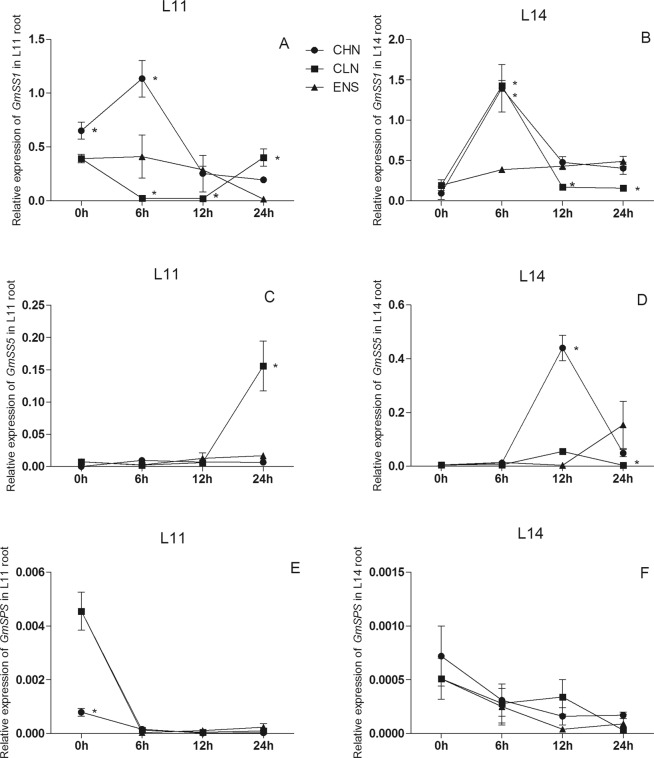
The expression of *GmSS1, GmSS5, GmSPS* in the root of CV. L11 (**A**,**C**) and CV. L14 (**B**,**D**). Values are the means of the three replicates. STD errors are the standard deviation of three replicates (p < 0.01). *Means at one sampling time, CHN or CLN treatment were significant higher/lower than ENS treatment.

The *GmSS1* expression in roots of both cultivars after 12 h of CHN and ENS treatments were higher than that of CLN treatment (P < 0.05) (Fig. 8A,B). The expression of *GmSS1* in CV.L11 root after 24 h of CLN treatment was higher than those of CHN and ENS treatments (Fig. 8A), while ENS and CHN treatments were higher than CLN treatment in the high yield CV.L14 (P < 0.05) (Fig. 8B). The *GmSS5* expression in CV.L11 root had no significant difference among CHN, CLN and ENS treatments, however, the *GmSS5* expression after 24 h of CLN treatment was significantly higher than those of CHN and ENS treatments (Fig. 8C), in the high yield CV.L14 root, there were no significant difference among CHN, CLN and ENS treatments after 0 h and 6 h (Fig. 8D). The *GmSS5* expression of CV.L14 root after 12 h of CHN treatment was significantly higher than those of CLN and ENS treatments (P < 0.05) (Fig. 8D). The expression of *GmSS5* after 24 h of ENS treatment was significantly higher than those of CHN and CLN treatments (P < 0.05) (Fig. 8D). Compared with *GmSPS* expression in leaf, there were lower expression of *GmSPS* in root. The *GmSPS* expression in CV.L11 root after 0 h of CLN treatment was lower than that of CHN treatment (Fig. 8E), however in the high yield CV.L14 root, there were no significant differences among CHN, CLN and ENS treatments (Fig. 8F). After 6 h, 12 h and 24 h, there were no significant differences of *GmSPS* expression among CHN, CLN and ENS treatments in the root of both cultivars (Fig. 8E,F).

## Discussion

Nitrogen is very important for the improvement of grain yield^36,37^. In plant, there is a close relation between nitrogen and carbon assimilation. In this research, all measured parameters had significant differences among three nitrogen treatments (Table 1). Under low nitrate condition, the nitrate accumulation in shoot of tobacco inhibited the root growth^38^. As the plant grew under a prolonged N limitation condition, the carbon requirement for organs was decreased, however, under the high N condition, plant required more carbon for nitrogen metbolism for plant growth^7^. Scheible *et al*.^33^ and Robinson *et al*.^39^ reported that more nitrate application promoted shoot growth. In this research, compared with CLN treatment, the shoot dry mass after 0 h of CHN treatment accumulated more biomass (Fig. 1A,B), while the root biomass had no significant differences (Fig. 1C,D). After enriched N treatment for 12 h, the root/shoot rate was lower than that of CLN, after enriched N treatment for 24 h, there were no significant differences between ENS and CLN treatments (Fig. 2A,B). The interaction of cultivar × treatment was not significant for root dry mass (Table 1). These results implied that, after enriched N at R1 growth stage, the shoot biomass increased more than that of root biomass. While at R1 growth stage, soybean demanded a plentiful nutritions and better canopy for photosynthesis, substance accumulation and material transportation^4,9,34^, the increased shoot biomass benefited for reproductive growth. Among three N treatments, the root/shoot rate of high yield cultivar was always lower than that of common yield cultivar, which implied that high yield cultivar have a strong root systems. After CLN and ENS treatments for 6 h, the shoot and root dry mass was significantly higher than those of 0 h, respectively. It indicated that, R1 stage is the period of rapid growth of soybean. After treatment for 6 h, there were no significant differences of shoot dry mass between ENS and CLN treatments of common yield cultivar, however, in high yield cultivar, the shoot dry mass of ENS treatment was significantly higher than that of CLN treatment. This was contrary to the root dry mass. After treatment for 6 h, the root dry mass of ENS treatment was significantly higher than that of CLN treatment of common yield cultivar, however, in high yield cultivar, there were no significant differences of shoot dry mass between ENS and CLN treatments. This implied that after N enrichment at R1 stage, the range of shoot growth in high yield cultivar was higher than root growth, the common yield cultivar was on the contrary.

**Table 1:** ANOVA of the parameters related with the biomass and sucrose metabolism in soybean shoot and root.

Traits	Source of variation						
	Cultivar(C)	Sampling time(S)	Nitrogen treatment(T)	C × S	C × T	S × T	C × S × T
Shoot dry mass(g/plant)	*	*	*	*	*	*	*
Root dry mass (g/plant)	*	*	*	*	NS	*	*
Biomass (g/plant)	*	*	*	*	*	*	*
Root/Shoot ratio	*	NS	*	NS	*	*	*
Shoot soluble sugar(%)	*	*	*	*	*	*	*
Root soluble sugar (%)	NS	*	*	*	*	*	*
Shoot sucrose (%)	NS	*	*	*	*	*	*
Root sucrose(%)	*	*	*	*	*	*	*
Shoot starch(mg/g)	*	*	*	*	*	*	*
Root starch(mg/g)	NS	*	*	*	*	*	*
Shoot SS activity	NS	*	*	*	*	*	*
Root SS activity	*	*	*	NS	*	*	*
Shoot SPS activity	*	*	*	*	*	*	*
Root SPS activity	*	*	*	*	*	*	*
Shoot *GmSPS*	*	*	*	*	*	*	*
Root *GmSPS*	*	*	*	*	*	*	*
Shoot *GmSS1*	*	*	*	*	*	*	*
Root *GmSS1*	*	*	*	*	*	*	*
Shoot *GmSS5*	NS	*	*	*	*	*	*
Root *GmSS5*	*	*	*	*	*	*	*

^*^Significant at the 0.05 probability levels. NS, Not significant.

Sucrose is the central of energy sustainability^40^. Under different nitrogen treatments, cultivars had no significant differences of root soluble sugar and starch content, and shoot sucrose content (Table 1). Sucrose primarily produced in leaf and it sometimes was resynthesized in root^40^. In this research, the contents of soluble sugar in shoot and root decreased rapidly after enriched N treatment at R1 growth stage. Kumari *et al*.^40^ and Sun *et al*.^41^ concluded that under N limitation condition, the decreased Rubisco was associated with the increased level of soluble sugar, the reduction of soluble sugar content may benefit for photosynthesis. The shoot sucrose content in CV.L14 of CLN treatment was always higher than that of CHN treatment (Fig. 3D), and the root sucrose content had the same tendency in both cultivars (Fig. 4C,D), this results consisted with Liu *et al*.^26^ that high nitrogen application was not conducive to sugar accumulation. After 24 hours of ENS treatment at R1 growth stage, the tendency of sucrose content was opposite with starch content in both shoot and root. This was consistent with Huber *et al*.^12^ that, it existed a negative correlation between the content of sucrose and starch.

**Figure 9:**
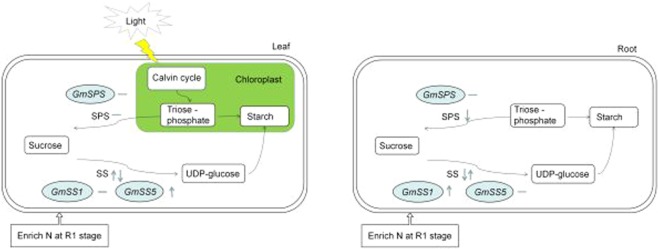
Rapid effect of enriched N at R1 stage on sucrose metabolism of soybean. Compared with CLN treatment, after enriched N at R1 stage, the SS activity of leaf was increased first and then decreased. However the SS activity of root was decreased first and then increased. The *GmSS5* expression in leaf and *GmSS1* expression in root was up-regulated. Means compared with CLN treatment, the enzyme activity and gene expression have no significant changes.

There was a positive correlation between SPS activity and sucrose content, but negatively correlated with starch content^41,42^. Liu *et al*.^26^ and Li *et al*.^25^ reported that, nitrogen decreased the activities of SS, it consisted with our result that after enriched N treatment for 6 h, but inconsistent with the enriched N treatment for 12 h (Fig. 5A,B). In this study, the results illustrated that under the enriched N treatment for 12 h, soybean plant tried to keep the balance of sucrose synthesis and decomposition in leaf (Fig. 9). Synthesising more sucrose would result in a higher biomass production^43,44^, and the plant also need to decompose sucrose into UDP-glucose and fructose for utilization in plant growth^25^, which implied that keeping the balance of sucrose synthesis and decomposition in leaf was benefit for plant growth. Many studies demonstrated that, the stress condition increased SPS activity and reduced the activity of sucrose hydrolytic enzymes^7,40^. Nitrate application inactivated the SPS and stimulated starch degradation, and then inhibited the root growth^45^. In this research of both cultivars, the SPS activity in the root of ENS treatment was reduced (Fig. 6C,D), which implied that the reduced SPS activity would result in the reduction of root/shoot ratio after the ENS treatment.

The change of sucrose content in different cultivars was related to the different levels of SS and SPS expression^46^. The over expression of *SPS* led to the increased sucrose/starch rate^32^. In cucumber, overexpression *CsSPS* led to carbon metabolism prioritizing sugar transport^22^. Suppressed *CsSPS* promoted carbon to starch synthesis^22^. While, the expression of *GmSPS* had no significant changes among CHN, CLN and ENS treatments, since the expression of *GmSPS* was necessary for sucrose accumulation but does not change during the regulation of sugar metabolism^46^. In high yield cultivar, the expression of *GmSS1* and SS activity got the same tendency, but in common yield cultivar, the high *GmSS1* expression did not cause high SS activity in root, this was consistent with Mizuno *et al*.^46^. As compared with the tendency of SS activity, *GmSS5* expression had a different tendency in common yield cultivar and high yield cultivar. Geigenberger *et al*.^11^ reported that, the expression of individual enzyme did not limit the sucrose convert into starch in starch accumulation pathway. The result indicated that, N application have different effects on SS genes expression, the different tendency of *GmSS1* and *GmSS5* gene expression in root implied that the different genes of one enzyme had different expressions, individual gene expression did not limit the activity of enzyme. In a short-term of enriched N at R1 stage, the transcript level of SS and SPS were changed, but did not limit the SS and SPS activities.

In conclusion, enriched N at R1 stage had a rapid effect on the transcript level of SS and SPS, but it did not influenced the SS and SPS activities. The appropriate sucrose synthesis and decomposition level in soybean benefited for the growth of soybean at R1 stage. Enriched N application at R1 stage suppressed rapidly the synthesis of the soluble sugar. There was a negative correlation between the content of sucrose and starch in shoot and root. In the high yield cultivar, the shoot growth was priority to root growth, the common yield cultivar was on the contrary. Under continuous N limitation, the root biomass accumulated firstly, then the shoot biomass. Enriched N at R1 stage improved the shoot biomass rapidly.

## Materials and Methods

### Plant material and culture conditions

Two soybean cultivars with different yield potentials, CV.Liaodou11 (L11) and CV.Liaodou14 (L14), were used. CV.L14 is a high yield cultivar with 4,923 kg/ha in 2000^47^. CV.L11 has a relatively low yield cultivar (about 3,000 kg/ha)^48^. The experimental pot field was at Shenyang Agricultural University, China (41°48′11.75″N and 123°25′31.18″E), with 22 °C average temperature, the average solar duration of 16 hours throughout the growing season. Seeds were rinsed with deionized water, and then sown in the pot filled with 7 kg silica sand. Each pot was thinned to 2 plants at cotyledon stage, and then 50% Hoagland solution^49^ was applied to each pot, the volume of watering was determined.

In this experiment, there were three N treatments as follows: continuous high nitrogen (CHN) treatment provided continuously high N (7.5 mM), continuous low nitrogen (CLN) treatment provided continuously low N (0.75 mM), enrich nitrogen supply at R1 stage (ENS) treatment provided continuously low N (0.75 mM) before R1 stage and then enriched nitrogen supply at R1 stage (7.5 mM). The high N (7.5 mM) was supplied as a mixture of 2 mM/L Ca(NO_3_)_2_·4H_2_O, 2.5 mM/L KNO_3_, 0.5 mM/L (NH_4_)_2_SO_4_, 0.5 mM/L KH_2_PO_4_, 1 mM/L MgSO_4_·7H_2_O, 0.005 mM/L KI, 0.1 mM/L H_3_BO_3_, 0.1 mM/L MnSO_4_·4H_2_O, 0.03 mM/L ZnSO_4_·7H_2_O, 0.001 mM/L NaMoO_4_·2H_2_O, 0.0001 mM/L CuSO_4_·5H_2_O, 0.0001 mM/L CoCl_2_·6H_2_O, 0.01 mM/L Na_2_-EDTA, 0.01 mM/L FeSO_4_·4H_2_O. And the low N (0.75 mM) was supplied as a mixture of 0.2 mM/L Ca(NO_3_)_2_·4H_2_O, 0.25 mM/L KNO_3_, 0.05 mM/L (NH_4_)_2_SO_4_, 0.5 mM/L KH_2_PO_4_, 1 mM/L MgSO_4_·7H_2_O, 0.005 mM/L KI, 0.1 mM/L H_3_BO_3_, 0.1 mM/L MnSO_4_·4H_2_O, 0.03 mM/L ZnSO_4_·7H_2_O, 0.001 mM/L NaMoO_4_·2H_2_O, 0.0001 mM/L CuSO_4_·5H_2_O, 0.0001 mM/L CoCl_2_·6H_2_O, 0.01 mM/L Na_2_-EDTA, 0.01 mM/L FeSO_4_·4H_2_O. The reduced elements of Ca^2+^, and K^+^ in the low nitrogen treatment was added with CaCl_2_ (1.8 mM/L) and K_2_SO_4_ (1.125 mM/L). Each cultivar of one treatment had 12 pots as replicates, each time sampled 3 pots as 3 replicates and the average of two plants in one pot was considered as one replicate.

### Plant sampling and measurements

After enriched nitrogen supply at R1 stage, the first harvest of each plant was carried out immediately (0 h), then harvested after 6 h, 12 h and 24 h of enriched nitrogen supply at R1 stage (ENS treatment), respectively. After sampled, roots were rinsed using distilled water. The uppermost fully expanded leaf and 2 g root tips were cut from each pot and immediately put into liquid nitrogen, and then stored at −80 °C for the measurements of enzyme activity and gene expression. The rest of plant samples were separated into above ground part (shoot) and root, and then oven-dried at 105 °C for 0.5 hours followed by 80 °C for 2 day.

### Determination of enzymes and sugar

Frozen samples were used to measure the activities of enzymes including sucrose synthetase and sucrose phosphate synthase according to the method of Liu *et al*.^50^ with slight modification. The extraction media containing 100 mM/L Tris-HCl (pH 7.2), 1 mM/L EDTA, 10 mM/L MgCl_2_, 10% polyvinylpyrrolidone (PVP) and 1 mM/L DTT^50,51^. Sample (0.3 g) was ground on ice in extraction buffer. The homogenate was centrifuged at 12, 000 × *g* for 15 min at 4 °C. The reaction buffer of SPS activity contained 12 mM/L UDP-glucose, 40 mM/L fructose-6-P, 200 mM/L Tris-HCl (pH 7.0), 40 mM/L MgCl_2_ and 200 μL extract. The reaction buffer of SS changed the 40 mM/L fructose-6-P of SPS reaction buffer into 40 mM/L sucrose. The reaction was initiated by incubating the enzyme at 30 °C for 30 min, and stopped using 100 μL 2 mol/L of NaOH. Then the solution was heated at 100 °C for 10 min. After cooling the solution, adding 1 mL of 0.1% (w/v) resorcin in 95% (v/v) ethanol and 3.5 mL of 30% (w/v) HCl, then incubated for 10 min at 80 °C. The SPS reaction liquid and SS reaction liquid were calculated from a standard curve measured at A_480_ nm and A_540_ nm, respectively.

The contents of soluble sugar, sucrose and starch were extracted by 0.1 g of ground sample, mixed with 80% (v/v) ethanol at 80 °C for 30 min, then centrifuged at 10,000 × *g* for 10 min. The residue was extracted in two more times using 80% ethanol. The three supernatants were combined and added by 80% ethanol to a total volume of 5 mL^52^. Samples of 1 mL supernatants were mixed with 5 mL of anthrone reagent, then incubated in a boiling water bath for 10 min. After the supernatant was cooled, the soluble sugar content was determined by spectrophotometry at A_620_ nm^53^. Samples of 0.4 mL supernatants mixed with 0.2 mL 2 M NaOH and incubated in a boiling water bath for 5 min. After cooling, the supernatant mixed with 2.8 mL 30% HCl and 0.8 mL 0.1% resorcinol, then reaction in 80 °C water bath for 10 min, the sucrose content was determined by spectrophotometry at A_480_ nm^53^. The ethanol-insoluble residue was used for starch extraction. After evaporation for ethanol remove, the samples were mixed with 2 mL distilled water at 100 °C for 15 min. Then 2 mL 9.2 mol/L HClO_4_ were added into samples for 15 min to hydrolyze starch. After that, added 4 mL distilled water into samples and centrifuged at 4000 × *g* for 10 min. The residue was extracted by 2 mL 4.6 mol/ L HClO_4_ once again. Then, two supernatants were combined and added by distilled water to a total volume of 20 mL. The content of starch was determined by spectrophotometry with anthrone reagent at A_620_ nm^54^.

### Genes expression

*GmSS1* and *GmSS5* encode SS, *GmSPS* encodes SPS. We obtained soybean genes that were homologous with *Arabidopsis* (https://www.arabidopsis.org/; https://phytozome.jgi.doe.gov/pz/portal.html). The information of selected genes and primers are in Table 2. Total RNA was isolated from sampled roots and leaf according to the Plant Total RNA Isolation Kit (Qiagen, Hilden, Germany) and using SuperScript III first-strand synthesis system for cDNA synthesis (Thermo Fisher Scientific, Waltham, MA, USA)^55^. RNA purity and concentration were measured by NanoDrop ultraviolet spectrophotometer (Thermo Fisher Scientific). After reverse transcription the expression of several genes were using the real-time PCR. A 15 µL reaction system was used for the qRT-PCR analysis, which consisted of 7.5 µL of SYBR qPCR mix, 1.2 µL PCR forward primer (10 µM), 1.2 µL PCR reverse primer (10 µM), 3 µL DNA (100 ng), and 2.1 µL dH_2_O. The qPCR protocol followed that described in Jia *et al*.^55^. Result were expressed as percentage of the constitutive *EF1ɑ* gene expression level^55^.

**Table 2:** Soybean gene locus, Arabidopsis homologues and qRT-PCR primer sequences.

Gene Name	Primer		Soybean Locus	Arabidopsis Locus	Amino Acid Identity
*GmSPS*	Forward (5′–3′)	ATGCTGGAGATAGTGCTGCT	Glyma.08G308600.1	AT1G04920.1	82.60%
	Reverse (5′–3 ′)	CCAAGGGAATGACCTGTCAGAA			
*GmSS1*	Forward (5′–3′)	GGTGTGCAATTCCTCAACCG	Glyma.13G114000.1	AT5G20830.1	91.70%
	Reverse (5′–3′)	CCAAAAGTGGGTGCAAGCTC			
*GmSS5*	Forward (5′–3′)	CCAAGCACCAGACCCAGTG	Glyma.14G209900.1	AT5G37180.1	81.50%
	Reverse (5′–3′)	CCAAGGACATCTGCTTGCCCA			

### Statistical analysis and calculations

The assays of dry matter, root/shoot ratio, enzymes and genes were determined in three biological replications, and the means and standard deviations were calculated. The ratio of root/shoot is the root dry matter divide shoot dry matter. Statistical analysis of three-way analysis of variance (ANOVA) and the least significant difference (LSD) test at the 0.05 level of confidence were accomplished using SPSS 17.0 (SPSS Inc., Chicago, IL, USA).
